# 

**Published:** 1872-07

**Authors:** 


### INDEX OF SUBJECTS.

### ORIGINAL COMMUNICATIONS.

Ovariotomy Case....................................................385
Fevers.............................................................389

### SELECTIONS.

On Functional Medicine.............................................394

### EXTRACTS AND GLEANINGS.

Bromide of Potassium for Hysteric Gastralgia Consequent upon Change of Life—Sulphurous Acid Lotion in the Treatment of Contused Wounds. 400; For Gastralgio—Stimulating Hypodermic Injections in Typhoid Fever, 401; For Leuchorrhœa—Pessary for Offensive Uterine Discharges, 402; Constant Galvanic Current in Treatment of Neuralgia, 403; Chloroform in Labor—Chloral in Urino-Genitary Diseases; 403; Preservation of Tinct. Kino from Gelatinizing, 404; For Menorrhagia—Chorea, 406; For Diarrhœa, 408; The Use of Iron in Scarlatina—Inunction of Newly Born Infants, 409; Hyperæsthesia of the Throat, 410; Ipecac in Dysentery, [illegible]; Hyperæsthesia of Stomach, 412; Laxative Mixture—Chilblains, 413; Hyperæsthesia of Testis, 414; Hyperæsthesia of the Heart, 415; New Operation for Cure of Varicose Veins, 416; Some of the Ill Effects of Bromide of Potassium, 417; Arsenic in Multiple Glands, 418; Treatment of Facial and Dental Neuralgia, 420; Is Phthisis Contagious? 421; Colic in Babies, 422; Nitrite of Amyl in Epilepsy, 425; Treatment of Physostigma, 426; Phosphorus for Sleeplessness, 427; Glycerine in Putrid Sore Throat, 428; Meat Extracts, 430.

### EDITORIALS, REVIEWS, ETC.

Notes on Our Visits to Colleges and Hospitals in Philadelphia..........432
Ponce De Leon Spring...................................................434
English "Good Taste"...................................................435
Suggestions and Kind Words from Friends................................436
Literary Notices—Periodicals...........................................442
Bibliographical........................................................443

